# Establishment of a Tetra‐Primer ARMS‐PCR Assay to Discriminate Genotypes I and II African Swine Fever Viruses

**DOI:** 10.1155/tbed/2248071

**Published:** 2026-05-14

**Authors:** Leilei Ding, Kun Xue, Tao Ren, Sihan Zhang, Weldu Tesfagaber, Zhenjiang Zhang, Feihu Guan, Jie Zhang, Yuanmao Zhu, Renqiang Liu, Fang Li, Zhigao Bu, Qianyi Zhang, Encheng Sun, Dongming Zhao

**Affiliations:** ^1^ State Key Laboratory of Animal Disease Control and Prevention, Professional Laboratory for African Swine Fever (Harbin), Harbin Veterinary Research Institute, Chinese Academy of Agricultural Sciences, Harbin, 150069, China, caas.cn; ^2^ College of Veterinary Medicine, Xinjiang Agricultural University, Urumqi, 830052, China, xjau.edu.cn; ^3^ Institute of Western Agriculture, The Chinese Academy of Agricultural Sciences, Changji, 831100, China, caas.cn; ^4^ SHIHEZI University, Shihezi, 832000, Xinjiang Uygur Autonomous Region, China, shzu.edu.cn; ^5^ China Institute of Veterinary Drug Control, Beijing, 100086, China

**Keywords:** ASFV, differential detection, genotype I and II, SNP, tetra-primer ARMS-PCR

## Abstract

The African swine fever virus (ASFV), particularly the genotypes I and II strains that have spread beyond Africa, continues to threaten global swine production and food security. Currently, African swine fever (ASF) control strategies rely heavily on biosecurity measures underpinned with molecular diagnostics. To address the need for genotyping, the present study developed a tetra‐primer amplification refractory mutation system PCR (ARMS‐PCR) assay capable of discriminating genotypes I and II ASFVs, as well as their mixed infections. The assay utilizes a multiplex of four primers: two outer universal primers and two allele‐specific inner primers targeting a conserved single nucleotide polymorphism (SNP, G1656A) of the *B646L* gene, specific to genotypes I and II ASFVs. The tetra‐primer ARMS‐PCR assay demonstrated a detection limit of 100 copies per reaction, which is comparable to that of the WOAH‐recommended PCR method, and no cross‐reactivity was observed with eight other porcine viruses. Furthermore, clinical validation confirmed that the assay demonstrated a 97.22% coincidence rate (35/36) with the standard WOAH‐recommended PCR method. In summary, the tetra‐primer ARMS‐PCR assay provides a practical, specific, and cost‐effective tool for ASF epidemiological surveillance, well‐suited for resource‐limited regions and those with cocirculation of genotypes I and II ASFVs.

## 1. Introduction

African swine fever (ASF), caused by the ASF virus (ASFV), is a devastating viral infectious disease affecting wild and domestic pigs. ASFV, the sole member of the *Asfarviridae* family, is a large, enveloped double‐stranded DNA virus with a 170–190 kb genome encoding 151–167 proteins [1]. Based on sequence variations within the 478‐nt C‐terminal region of the *B646L* gene (encoding the p72 protein), ASFVs are classified into at least 24 genotypes [2]. While all of these genotypes circulate within Africa, only genotypes I and II have spread beyond the continent.

Since its first description in Kenya in 1921, ASFV has remained endemic in Africa [3]. In 1957, genotype I ASFV first emerged in Portugal [4], which subsequently spread to Europe, South America, and the Caribbean and was eventually eradicated worldwide by the mid‐1990s, with the exception of the Italian island of Sardinia [5]. The second global wave began in 2007 when genotype II ASFV first emerged in Georgia and subsequently spread to Europe, Asia, and the Americas, heralding a new transmission era [6–9]. However, a re‐emergence of genotype I ASFV occurred in China in 2021 [10]. Notably, in 2023, genotype I/II recombinant viruses have been reported successively in China [11], Vietnam [12], and Russia [13]. Despite genotype II viruses continued dominance as the predominant global strain, genotype I viruses, particularly genotype I/II recombinant virus, present a growing concern due to their ability to evade the immune protection conferred by genotype II‐based live attenuated vaccines [11, 14]. The ongoing global spread of ASF, now documented in 68 countries from January 2022 to August 2025 across Africa, Europe, Asia, and the Americas, represents a profound and persistent threat to international pork production and food security (https://www.woah.org).

Currently, PCR‐based sequencing targeting the C‐terminal end of the *B646L* gene is considered the gold standard for ASFV genotyping, which is performed by using the primers P72‐U and P72‐D [15]. Other approaches include high‐throughput sequencing (HTS) platforms that target the complete genome [16] or genes [17], a suspension microarray comprising 52 probes (based on xMAP technology) to target single nucleotide polymorphisms (SNPs) in the C‐terminal end of the *B646L* gene for 22 genotypes [18], and multiplex quantitative PCR (qPCR) assays that target SNPs or different genes to differentiate genotypes I and II ASFVs [19, 20]. However, these assays often require expensive equipment, fluorescent probes, specialized reagents, and trained personnel, limiting their use in resource‐constrained settings.

Single nucleotide polymorphism (SNP), characterized by a single‐base variation at a specific genomic position between individuals have served as the most valuable molecular marker for research and applications, including genotyping and allele discrimination, gene mapping and cloning, the analysis of evolutionary conservation, and molecular breeding in agriculture [21]. Among the SNP‐detection methods, tetra‐primer amplification refractory mutation system PCR (ARMS–PCR) offers a simple, rapid, and cost‐effective alternative [22]. This assay exploits the principles of tetra‐primer PCR [23] and the Amplification Refractory Mutation System (ARMS) [24], both of which are derived from a mismatch strategy to produce an allele‐specific reaction. This allele‐specific approach discriminates between SNP alleles using primers with a deliberate 3′ terminal mismatch. PCR amplification is critically dependent on a perfectly matched 3′ end, ensuring that extension occurs only for the complementary allele. Tetra‐primer ARMS‐PCR uses four primers, including a pair of nonallele‐specific primers (outer primers) and a pair of allele‐specific primers (inner primers) [22]. Designing the outer primers at varying distances from the SNP enables the clear distinction of allele‐specific fragments by size via agarose gel electrophoresis. Owing to its advantages of being simple, rapid, reliable, and cost‐effective, the Tetra‐primer ARMS‐PCR assay has been widely adopted for SNP detection and genotyping [25–27].

Here, we developed a tetra‐primer ARMS‐PCR assay based on a conserved SNP of the ASFV *B646L* genotyping region to differentiate genotype I, genotype II, and mixed infections. Moreover, we evaluated the assay’s analytical sensitivity using standardized plasmids and assessed its performance on clinical samples in comparison with the WOAH‐recommended PCR method.

## 2. Materials and Methods

### 2.1. Viruses and Plasmids

The ASFV strains used in this study include genotype I viruses (SD/DY‐I/21, HeN/ZZ‐P1/21, JS/LG/21, HeN123014/22, and IM/DQDM/22) [10, 11] and genotype II viruses (HLJ/18, HuB/628/20, JL/1/20, HLJ/HRB1/20, HuB/1/20, and HeB/Q3/20) [28, 29], all viruses are isolated in the Harbin Veterinary Research Institute (HVRI). In addition, virus stocks of classical swine fever virus (CSFV), porcine reproductive and respiratory syndrome virus (PRRSV), porcine epidemic diarrhea virus (PEDV), pseudorabies virus (PRV), porcine transmissible gastroenteritis virus (TGEV), porcine rotavirus (PoRV), porcine circovirus 2 (PCV2), and porcine circovirus 3 (PCV3) were maintained at the HVRI, Chinese Academy of Agricultural Sciences. These viral stocks were stored at −80°C. Recombinant plasmids pCAGGS‐I and pCAGGS‐II, which contain the *B646L* gene of genotypes I and II ASFVs, respectively, were constructed and stored at −20°C in HVRI [19].

### 2.2. Clinical Samples and Nucleic Acid Extraction

Clinical samples (blood, oral and rectal swabs, spleen, tonsil, and lymph node) were processed as described previously [19]. Viral genomic DNA/RNA was then extracted from 200 μL of either virus stocks or processed clinical sample supernatants using the TIANamp Virus DNA/RNA kit (TIANGEN, Beijing, China) according to the manufacturer’s instructions.

### 2.3. Primers Design

Based on the SNP at position 1656 in the *B646L* gene of the genotypes I and II ASFVs, two allele‐specific primers (inner primers) were designed using a web‐based tool https://primer1.soton.ac.uk/primer1.html [22, 30]. The universal typing primers P72‐U and P72‐D were used as the outer primers [15]. For comparative analysis, the PPA‐1 and PPA‐2 primers from the WOAH‐recommended PCR assay were synthesized [31]. The primers used in the study were commercially synthesized by Shihe Biotech Co. Ltd. (Harbin, China) and are listed in Table 1.

**Table 1:** Specific primers targeting the *B646L* gene in the study.

Division	Primer name		Sequence (5′→3′)	TM (°C)	Amplicon (bp)	References
Tetra‐primer ARMS‐PCR	Inner forward	F1	CTG CTC ATG GTA TCA ATC TTA TCG ATC AG	59.62	310	—
		F2	CTG CTC ATG GTA TCA ATC TTA TCG ATT AG	58.21		
		F3	CTG CTC ATG GTA TCA ATC TTA TCG ATG AG	59.62		
	Inner reverse	R1	GAG CTG CAG AAC TTT GAT GGA TAT	56.15	220	—
		R2	GAG CTG CAG AAC TTT GAT GGA CAT	57.86		
		R3	GAG CTG CAG AAC TTT GAT GGA GAT	57.86		
	Outer forward	P72‐U	GGC ACA AGT TCG GAC ATG T	55.16	478	[15]
	Outer reverse	P72‐D	GTA CTG TAA CGC AGC ACA G	55.16		
PCR recommended by WOAH	PPA‐1		AGT TAT GGG AAA CCC GAC CC	57.45	257	[31]
	PPA‐2		CCC TGA ATC GGA GCA TCC T	57.32		

*Note:*—, no relevant content.

### 2.4. Optimization of the Tetra‐Primer ARMS‐PCR Conditions

To optimize the tetra‐primer ARMS‐PCR assay, we utilized two recombinant plasmids (pCAGGS‐I and pCAGGS‐II) as templates. The tetra‐primer ARMS‐PCR reaction system is 25 μL: 2 × Premix Taq (TaKaRa; Cat.# RR901) of 12.5 μL. Reaction conditions were optimized by testing nine sets of primers (U/D/F1/R1, U/D/F1/R2, U/D/F1/R3, U/D/F2/R1, U/D/F2/R2, U/D/F2/R3, U/D/F3/R1, U/D/F3/R2, and U/D/F3/R3), five annealing temperature (48, 50, 52, 54, and 56°C) and three concentrations of primers (0.4/0.4/0.4/0.4, 0.8/0.8/0.4/0.4, and 0.8/0.8/0.8/0.8 pmol/μL), to determine the optimal primers, annealing temperature and primer concentration. The PCR cycling consisted of an initial denaturation at 95°C for 5 min, followed by 35 amplification cycles at 95°C for 30 s, 52°C for 30 s, and 72°C for 30 s, with a final extension step at 72°C for 10 min. Subsequently, the PCR products were subjected to electrophoresis through a 1% agarose gel in 1 × TAE buffer (Beijing Lanjieke Technology Co., Ltd., China) and stained with ethidium bromide to visualize using an Amersham Imager 680 (GE Healthcare, USA).

### 2.5. Validation and Analytic Specificity of the Tetra‐Primer ARMS‐PCR Assay

To conduct a preliminary evaluation of the tetra‐primer ARMS‐PCR assay, the viral DNA from genotype I ASFVs (SD/DY‐I/21, HeN/ZZ‐P1/21, JS/LG/21, HeN123014/22, and IM/DQDM/22) and genotype II viruses (HLJ/18, HuB/628/20, JL/1/20, HLJ/HRB1/20, HuB/1/20, and HeB/Q3/20) were used as templates.

To determine the specificity of the tetra‐primer ARMS‐PCR assay, the nucleic acid from eight other swine viruses, including PRRSV, CSFV, PRV, PCV2, PCV3, PEDV, TGEV, and PoRV was used as a template.

### 2.6. Analytical Sensitivity of the Tetra‐Primer ARMS‐PCR Assay

The standard plasmids pCAGGS‐I and pCAGGS‐II were prepared as described previously [19]. Their copy number concentration was determined using the formula: *y* (copies/μL) = (6.02 × 10^23^) × (*x*(ng/μL) × 10^−9^ DNA)/(DNA length × 660). Based on this, the plasmids were serially diluted 10‐fold from 10^6^ to 10^0^ copies/5μL to evaluate their analytical sensitivity. This was directly compared to the sensitivity of the WOAH‐recommended PCR assay [31].

### 2.7. Comparative Validation of the Tetra‐Primer ARMS‐PCR and the WOAH‐Recommended PCR Assays From Clinical Samples

Viral DNA was extracted from 36 different types of clinical samples obtained from pigs infected with either genotypes I or II ASFV. These DNA extracts were ASFV positive tested by the WOAH‐recommended qPCR assay in our previous study [19]. In parallel, they were tested using the developed tetra‐primer ARMS‐PCR and WOAH‐recommended PCR [31] assays.

## 3. Results

### 3.1. Design of the Tetra‐Primer ARMS‐PCR for Differentiating Genotypes I and II ASFVs

**Figure 1:**
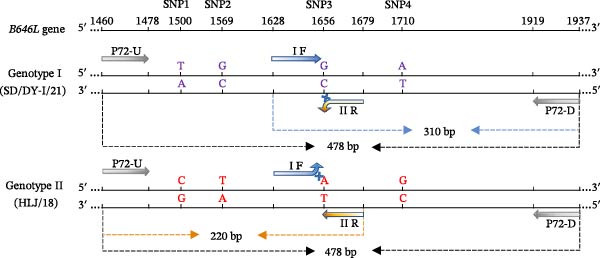
Schematic diagram of the tetra‐primer ARMS‐PCR for differentiating genotypes I and II ASFVs. Four conserved SNPs at positions 1500, 1569, 1656, and 1710 were identified by analyzing the complete or partial *B646L* genes of 126 genotype I and II ASFVs. The SNP3 (G1656A) at position 1656 was selected for the development of the tetra‐primer ARMS‐PCR assay. The P72‐U and P72‐D primers (the universal genotyping primers) served as the outer primers and amplified a 478 bp fragment common to all ASFVs. The I‐F and II‐R primers served as the allele‐specific inner primers. A 310 bp fragment was generated from genotype I ASFV, and a 220 bp fragment was generated from genotype II ASFV.

Previously, by analyzing the complete or partial *B646L* genes of 126 ASFV isolates (78 for genotype I and 48 for genotype II), we identified four conserved SNPs at positions 1500, 1569, 1656, and 1710 within the C‐terminal region of the gene [19]. In this study, we developed a tetra‐primer ARMS‐PCR assay using the established universal genotyping primers P72‐U and P72‐D as the outer primers. Simultaneously, the two allele‐specific primers (I F and II R) targeting the SNP at position 1656 were designed to serve as the inner primers. The outer primers amplified a 478 bp fragment common to all ASFVs (Figure 1). Genotype‐specific amplification was achieved with inner primers: a 310 bp fragment was generated from genotype I templates using the I‐F (inner) and P72‐D (outer) primer pair, while a 220 bp fragment was generated from genotype II templates using the P72‐U (outer) and II‐R (inner) primer pair (Figure 1). For samples with mixed genotype I and II infection, the three characteristic fragments (478, 310, and 220 bp) were simultaneously amplified.

### 3.2. Optimization of the Tetra‐Primer ARMS‐PCR Reaction Conditions

**Figure 2:**
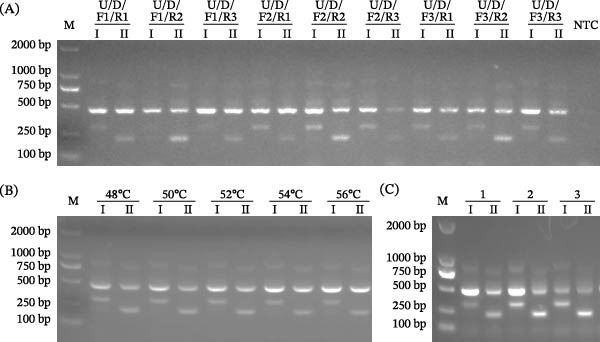
Optimization of the tetra‐primer ARMS‐PCR reaction system. The standard plasmids of pCAGGS‐I (genotype I) and pCAGGS‐II (genotype II) were used as templates to refine sets of primers (A), annealing temperature (B), and concentrations of primers (C). I, pCAGGS‐I. II, pCAGGS‐II. M, DNA marker. NTC, no‐template control. U, P72‐U. D, P72‐D. 1, 2, and 3 in C correspond to the concentrations (in pmol/μL) of primers U, D, F2, and R2, as follows: 1 (0.4, 0.4, 0.4, 0.4); 2 (0.8, 0.8, 0.4, 0.4); 3 (0.8, 0.8, 0.8, 0.8).

Reaction parameters for the tetra‐primer ARMS‐PCR assay were optimized using the standard plasmids of pCAGGS‐I and pCAGGS‐II as templates. As shown in Figure 2A–C, optimal amplification was achieved with primer set U/D/F2/R2 at an annealing temperature of 52°C and primer concentrations of 0.8, 0.8, 0.4, and 0.4 pmol/μL for primers P72‐U, P72‐D, F2, and R2, respectively. These amplification conditions were used for all subsequent tetra‐primer ARMS‐PCR reactions in this study.

### 3.3. Validation and Analytic Specificity Determination of the Tetra‐Primer ARMS‐PCR Assay

**Figure 3:**
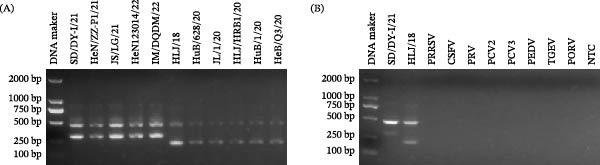
Validation and specific detection of the tetra‐primer ARMS‐PCR assay. (A) Preliminary validation. The assay was tested on genotype I strains (SD/DY‐I/21, HeN/ZZ‐P1/21, JS/LG/21, HeN123014/22, IM/DQDM/22) and genotype II strains (HLJ/18, HuB/628/20, JL/1/20, HLJ/HRB1/20, HuB/1/20, and HeB/Q3/20). (B) Specificity testing. The assay was challenged with genotype I ASFV (SD/DY‐I/21), genotype II ASFV (HLJ/18), PRRSV, CSFV, PRV, PCV2, PCV3, PEDV, TGEV, PoRV, and no‐template control (NTC).

The tetra‐primer ARMS‐PCR assay successfully differentiated between genotypes I and II ASFVs, generating two distinct fragment profiles (Figure 3A). All tested genotype I strains (SD/DY‐I/21, HeN/ZZ‐P1/21, JS/LG/21, HeN123014/22, and IM/DQDM/22) produced 478 bp and 310 bp fragments, and all genotype II viruses (HLJ/18, HuB/628/20, JL/1/20, HLJ/HRB1/20, HuB/1/20, and HeB/Q3/20) produced 478 bp and 220 bp fragments.

In the specificity analysis, the tetra‐primer ARMS‐PCR assay amplified DNA from both genotypes I and II ASFVs but not from eight other swine viruses, including PRRSV, CSFV, PRV, PCV2, PCV3, PEDV, TGEV, and PoRV (Figure 3B).

### 3.4. Comparative Analytical Sensitivity of the Tetra‐Primer ARMS‐PCR and WOAH‐Recommended PCR Assays

**Figure 4:**
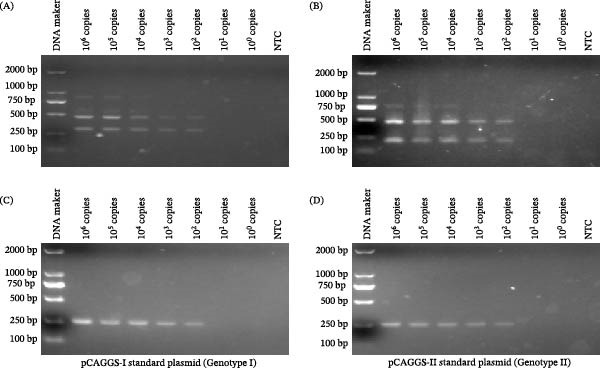
Analytical sensitivity of the tetra‐primer ARMS‐PCR assay compared to the WOAH‐recommended PCR method. Tenfold serial dilutions (10^6^ to 10^0^ copies per 5 μL) of the standard plasmids of pCAGGS‐I (genotype I) and pCAGGS‐II (genotype II) were detected by (A,B) tetra‐primer ARMS‐PCR and (C,D) WOAH‐recommended PCR, respectively. NTC, the no‐template control.

The tetra‐primer ARMS‐PCR assay detected a minimum of 100 copies of standard plasmids of pCAGGS‐I (genotype I ASFV) and pCAGGS‐II (genotype II ASFV) (Figure 4A,B). This level of sensitivity is comparable to that of the WOAH‐recommended PCR method using PPA‐1/2 primers [31], with both assays having a limit of detection (LOD) of 100 copies per reaction (Figure 4A–D).

### 3.5. Comparative Validation of the Tetra‐Primer ARMS‐PCR and WOAH‐Recommended PCR Assays Using Genotype I and II ASFVs Clinical Samples

**Figure 5:**
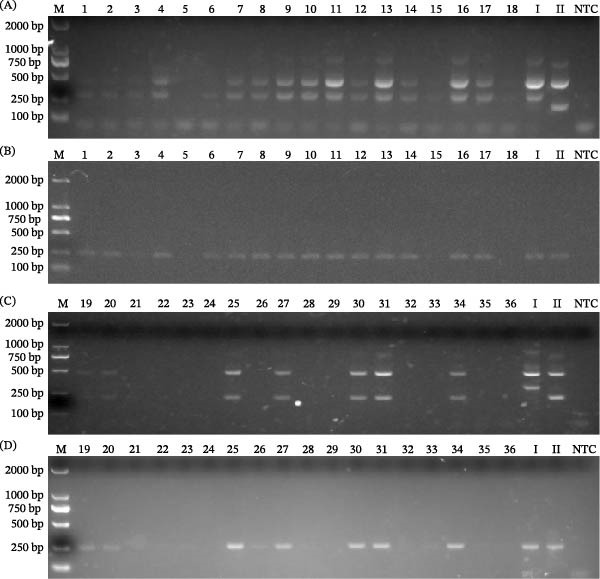
Validation of the tetra‐primer ARMS‐PCR assay for differential detection of genotype I and II ASFVs in clinical samples. Clinical samples of genotypes I and II ASFVs were detected by (A,C) Tetra‐primer ARMS‐PCR and (B,D) WOAH‐recommended PCR, respectively. Lanes 1, 2, 19, 20, blood; lanes 3, 4, 21, 22, oral swab; lanes 5, 6, 23, 24, rectal swab; lanes 7, 8, 25, 26, spleen; lanes 9, 10, 27, 28, tonsil; lanes 11, 12, 29, 30, lymph node; lanes 13, 14, 15, SD/DY‐I/21; lanes 16, 17, 18, HeN/ZZ‐P1/21; lanes 31, 32, 33, HLJ/18; lanes 34, 35, 36, HLJ/HRB1/20. I, pCAGGS‐I standard plasmid (genotype I). II, pCAGGS‐II standard plasmid (genotype II). NTC, no‐template control.

The Tetra‐primer ARMS‐PCR assay and the WOAH‐recommended PCR method were compared and validated using 36 clinical ASFV‐positive samples (18 for genotype I and 18 for genotype II) in a previous study [19]. For the 18 genotype I ASFV samples, the two methods yielded identical results (100% agreement). 15 were positive by the tetra‐primer ARMS‐PCR and WOAH‐recommended PCR assays (Figure 5A,B), and these positives showed Ct values of 20.61–32.41 via the WOAH‐recommended qPCR [19]. The remaining 3 samples, which were negative by both assays, showed high Ct values (33.28, 35.22, and 36.91) by the WOAH‐recommended qPCR.

For the 18 genotype II ASFV samples, 7 were positive by the tetra‐primer ARMS‐PCR and WOAH‐recommended PCR methods, and 10 were negative by both methods, yielding an overall agreement of 94.4% (17/18; Figure 5C,D). The 7 concordant positive samples exhibited Ct values ranging from 19.58 to 29.90, whereas the single discrepant sample had a Ct value of 34.48 via the WOAH‐recommended qPCR [19]. The 10 samples, which were negative by both the tetra‐primer ARMS‐PCR and the WOAH‐recommended PCR, yielded Ct values ranging from 32.73 to 38.47 by the WOAH‐recommended qPCR.

## 4. Discussion

This study established a tetra‐primer ARMS‐PCR assay based on the SNP at position 1656 of the ASFV *B646L* (p72) gene. The assay enables simultaneous differentiation between genotypes I, II, and mixed infections in a single PCR reaction using four primers. Given that only genotypes I and II ASFVs have spread beyond Africa, this assay is largely sufficient to meet the current demand for ASFV genotyping outside the African continent and is particularly valuable in regions where these two genotypes cocirculate.

The assay demonstrated a LOD of 100 copies per reaction for both pCAGGS‐I and pCAGGS‐II standard plasmids, a sensitivity comparable to the WOAH‐recommended PCR assay [31]. While it was slightly lower than an updated universal ASFV PCR assay (LOD: 60 copies) [32], and tenfold lower than the dual ARMS‐qPCR assay designed for genotype discrimination [19]. In addition, the tetra‐primer ARMS‐PCR assay successfully and simultaneously differentiates genotypes I and II ASFVs in a variety of clinical samples, including blood, swabs, or tissues. Notably, owing to the intrinsic sensitivity limit of the PCR technique itself, accurate detection with the tetra‐primer ARMS‐PCR assay was confined to samples exhibiting Ct values less than 30, whereas conventional PCR detection becomes unstable for samples with Ct values above 30. According to the research by Medrano and de Oliveira [33], the melting temperature, DNA quality, reagent equilibrium (particularly MgCl_2_ concentration), and primer balancing are critical factors for developing the tetra‐primer ARMS‐PCR assay. Consequently, future optimization of PCR reagent and primer concentrations may enhance the assay’s sensitivity and reduce nonspecific amplification. However, it is important to note that the tetra‐primer ARMS‐PCR assay is not suitable for SNPs in GC‐rich regions or for use with unpurified DNA samples [22, 33].

The *B646L* gene, the typing gene of ASFV, which encodes the highly immunogenic and antigenic major capsid p72 protein, is one of the most widely used diagnostic targets and is specifically utilized in the WOAH‐recommended PCR [31] and qPCR [34] assays. The tetra‐primer ARMS‐PCR assay used the universal typing primers P72‐U and P72‐D as outer primers [15], amplifying a 478 bp fragment common to nearly all ASFV genotypes. However, the specificity of the inner primers for genotypes I and II ASFVs requires further evaluation against other genotype viruses. Additionally, previous studies have reported that nine point mutations (C36T, G330A, C458T, G806A, G1175A, C1242T, A1713T, G1836A, and G1860A) have occurred in the B646L gene of ASFV in China [35]; therefore, the match between the tetra‐primer ARMS‐PCR primers and their target sequences in genotypes I and II viruses requires ongoing surveillance.

To date, the prevention and control of ASF rely primarily on early detection, strict animal quarantine, and culling procedures. Although qPCR is the WOAH‐recommended gold standard for the molecular diagnosis of ASF, conventional PCR remains highly useful, offering wider applicability in resource‐limited laboratories lacking sophisticated equipment or specialized personnel. Therefore, the tetra‐primer ARMS‐PCR assay is well suited for deployment in resource‐limited regions.

## 5. Conclusion

The tetra‐primer ARMS‐PCR assay offers a rapid, specific, and accurate tool for the current epidemiological monitoring of ASF, especially in regions with cocirculation of both genotypes I and II ASFVs.

## Author Contributions

Dongming Zhao, Encheng Sun, Qianyi Zhang, and Leilei Ding collaboratively conceived and designed the experiments. Leilei Ding, Kun Xue, Tao Ren, Sihan Zhang, Feihu Guan, and Jie Zhang performed experiments, collected the data and prepared the graphs. Dongming Zhao, Encheng Sun, Leilei Ding, Renqiang Liu, and Zhenjiang Zhang obtained research funding. Leilei Ding, Tao Ren, and Qianyi Zhang wrote the original draft of the manuscript. Dongming Zhao, Encheng Sun, Weldu Tesfagaber, and Zhigao Bu reviewed the manuscript, provided critical feedback, and edited the final text. Yuanmao Zhu and Fang Li provided essential experimental materials.

## Funding

This research was supported by the Shanghai Agriculture Applied Technology Development Program, China (Grant X2024‐02‐08‐00‐12‐F00049), the National Key R&D Program of China (Grant 2021YFD1800101), the Central Public‐interest Scientific Institution Basal Research Fund (Grant CAAS‐ZDRW202409), the Innovation Program of the Chinese Academy of Agricultural Sciences (Grant CAAS‐CSLPDCP‐202301), the China Postdoctoral Science Foundation (Grants 2024M753566 and 2024M763617), the China‐Burundi Arthropod‐transmitted Animal Disease Prevention and Control, and One Health Joint Laboratory (Grant 2023YFE0126200), and the Heilongjiang Provincial Natural Science Foundation of China (Grant JQ 2023C005).

## Disclosure

All authors have read and approved the final manuscript.

## Ethics Statement

All experiments with infectious ASFV were conducted in the enhanced biosafety level 3 (P3+) facility in the HVRI of the Chinese Academy of Agricultural Sciences (CAAS), which are approved by the Ministry of Agriculture and Rural Affairs (MARA) and the Animal Experimentation and Laboratory Animal Welfare Committee of HVRI, under license 220916‐01‐GJ.

## Conflicts of Interest

The authors declare no conflicts of interest.

## Data Availability

The data that support the findings of this study are available from the corresponding author upon reasonable request.
